# Do blood eosinophils strictly reflect airway inflammation in COPD? Comparison with asthmatic patients

**DOI:** 10.1186/s12931-019-1111-1

**Published:** 2019-07-10

**Authors:** Patrizia Pignatti, Dina Visca, Francesca Cherubino, Elisabetta Zampogna, Etienne Lucini, Laura Saderi, Giovanni Sotgiu, Antonio Spanevello

**Affiliations:** 1 Allergy and Immunology Unit, Istituti Clinici Scientifici Maugeri, IRCCS, Pavia, Italy; 2 Division of Pulmonary Rehabilitation, Istituti Clinici Scientifici Maugeri, IRCCS, Tradate, Italy and Department of Medicine and Surgery, Respiratory Diseases, University of Insubria, Varese-Como, Italy; 3 Division of Pulmonary Rehabilitation, Istituti Clinici Scientifici Maugeri, IRCCS , Tradate, Italy; 4 Clinical Epidemiology and Medical Statistics Unit, Department of Biomedical Sciences, University of Sassari, Sassari, Italy

**Keywords:** Airway inflammation, Sputum, Eosinophils, Asthma, COPD, Correlation

## Abstract

**Introduction:**

Airway eosinophilic inflammation is a characteristic of asthmatic patients and of a sub group of COPD subjects. Blood eosinophils are deemed as a good surrogate marker of sputum eosinophilic inflammation; however, controversial data have been published particularly in COPD. The aim of our study was to compare blood and sputum eosinophils in COPD and asthmatic patients in “real life”.

**Methods:**

Sputum was induced in stable patients with COPD or asthma with hypertonic saline solution and blood eosinophils were evaluated. Frequency of comorbidities was recorded. Correlations were performed stratifying patients by disease and comorbidities.

**Results:**

146 patients, 57 with COPD and 89 with asthma were evaluated. Blood and sputum eosinophils expressed as percentages were correlated in COPD (rho = 0.40; *p* = 0.004), but the entity of correlation was lower compared with asthmatic subjects (rho = 0.71; *p* < 0.0001). When blood eosinophils were expressed as counts the correlation was slightly lower than when expressed as percentages in COPD (rho = 0.35; *p* = 0.01) and in asthmatic patients (rho = 0.68; p < 0.0001). In COPD patients older than 73 years or with blood eosinophils higher than the median value (210.6 eos/μl), or co-diagnosed with hypertension, ischemic heart disease or atrial fibrillation no correlation between blood and sputum eosinophils was found.

However, the effect of ischemic heart disease and atrial fibrillation could be driven by hypertension since most of these patients have this comorbidity.

**Conclusion:**

Blood eosinophils correlated with sputum eosinophils to a lesser degree in COPD than in asthmatic patients. Older age, high blood eosinophils and hypertension affected the correlation between blood and sputum eosinophils, more studies are needed to evaluate the role of other cardiac comobidities.

## Background

In the last decades, characterization of airway inflammation highlighted the presence of different phenotypes not only in asthmatic but also in COPD patients [1, 2]. The old dichotomy of eosinophilic inflammation for asthma and neutrophilic inflammation for COPD was revised with a neutrophilic phenotype present in asthmatic patients, and eosinophilic inflammation in a subgroup of COPD subjects. Induced sputum is the non-invasive and reproducible [3] methodology most frequently used to assess airway inflammation but, since the methodology is not available in every clinical setting, the possibility to predict the eosinophilic phenotype through blood eosinophils represents a good opportunity and is widely used both in asthma and in COPD. Correlation between eosinophils in blood and airways depends on their recruitment from the bone marrow to blood and then to the tissue, triggered by inflammatory stimuli.

In asthmatic patients, the correlation is reliable, even if influenced by comorbidities (e.g., nasal polyposis) [4]. In COPD patients, blood eosinophils do not correlate with airway eosinophils evaluated through biopsies [5], whereas findings from induced sputum-based studies are inconclusive. Appropriate selection of COPD patients with an eosinophilic pattern permits to add inhaled corticosteroids to bronchodilators in the right patients limiting the risk of pneumonia and to select potential patients to be treated with new monoclonal antibodies against IL-5.

The aim of our study was to assess the correlation between blood and sputum eosinophils in COPD patients in “real life” and to compare this correlation with that found in asthmatic patients. Effect of age and comorbidities on blood and sputum eosinophils correlation was considered. To the best of our knowledge, this is the first study evaluating this correlation simultaneously in these groups of patients. Preliminary data were presented as abstract [6].

## Material and methods

### Patients

We evaluated COPD and asthmatic patients who underwent induced sputum to assess airway inflammation and blood eosinophil count in our Institute from November 2016 to August 2018.

COPD was diagnosed according to Global Initiative for Chronic Obstructive Lung Disease (GOLD) criteria [7]. Lung impairment was detected through the value of FEV_1_ (forced expiratory volume in the first second) post bronchodilator test and classified with the GOLD step from 1 to 4. No patients with step 4 underwent sputum induction. In addition, we clinically stratified COPD patients in a scale from A to D taking into account dyspnoea and number of exacerbations [7].

All patients had COPD without history of asthma or other allergic diseases. Asthma was diagnosed according to current Global Initiative for Asthma (GINA) guidelines [8].

None of the patients had infections of the upper respiratory tract or exacerbations in the previous 2 months of the enrolment. This study conformed to the declaration of Helsinki and was approved by the IRB of Istituti Clinici Scientifici Maugeri (number 2209 CE).

### Sputum induction and processing

Sputum was induced by inhalation of hypertonic saline aerosol. Briefly, 10 min after salbutamol inhalation (200 μg), hypertonic saline (4.5%), nebulized by an ultrasonic nebulizer (ULTRA-NEB 3000, DeVilbiss Healthcare Inc., Somerset, USA), was inhaled over four different time periods and then the patient was invited to cough and sputum was collected. FEV_1_ was monitored before and after each inhalation to either prevent or detect possible bronchoconstriction (Pony FX Spirometer, Cosmed, Chicago, IL, USA). After collection, the sputum sample was processed within 2 hours, according to the International Guidelines standardized method with dithiothreitol [8], and then centrifuged at 1000 x g for 5 min. The cell pellet was suspended in a volume of phosphate-buffered saline (PBS) equal to that of the filtered suspension. The total cell count was determined by a Burker chamber. The cell suspension was then centrifuged at 450 rpm for 6 min (Shandon 3 Cytocentrifuge; Shandon Southern Instruments, Sewickley, PA). Two cytospin slides were stained with Diff-Quick solutions (Medion Diagnostics AG Düdingen, Switzerland) for differential cell count. Sputum eosinophilia was defined when a percentage of sputum eosinophils > 3% occurred [9].

### Blood cell count

Bloodwas collected in K2EDTA tubes (Vacutainer, Becton Dickinson, Plymouth, UK). Peripheral blood eosinophils were determined using UniCelDxH 800 haematologyanalyser (Beckman Coulter, Pasadena, CA) for cell differentiation.

### Statistical analysis

An ad hoc electronic database was created to collect all study variables. Qualitative data were summarized with absolute and relative frequencies. Mean and standard deviation (SD) or median and interquartile range (IQR) were used for quantitative variables with a parametric and non-parametric distribution, respectively. Chi-squared or Fisher exact test was used to detect any statistical differences for qualitative variables. Student’s t and Mann-Whitney tests were used for quantitative variables following their parametric and non-parametric distribution, respectively. Spearman’s correlation was used to assess the relationship between eosinophils in the peripheral blood and in sputa. *P*-value less than 0.05 was considered statistically significant. Stata 15 statistical software was used for every statistical computation.

## Results

### Patients

We evaluated 146 patients, 57 with COPD (mainly step 2, A and B) and 89 with asthma (mainly moderate/severe). The characteristics of the enrolled subjects are shown in Table 1. Comorbidities of the enrolled subjects are shown in Table 2: 21.3% of COPD patients had one comorbidity, 23.4% two, 25.5% three, 19.1% four, 8.5% five and 2.1% six comorbidities. In COPD patients ischemic heart disease, atrial fibrillation and hypertension were more frequent than in asthmatic patients (*P* < 0.0001, *P* = 0.03 and P = 0.03, respectively).

**Table 1:** Characteristics of enrolled subjects

Variables	COPD (*n* = 57)	Asthma (*n* = 89)	*P*-values
Male, n (%)	43 (75.4)	43 (48.3)	0.001
Median (IQR) age, years	73 (66–77)	59 (51–69)	< 0.0001
Median (IQR) BMI, kg/m2	27 (24.0–30.8)	26 (23.0–29.4)	0.09
Median (IQR) pack-year	38 (23–50)	0 (0–10)	< 0.0001
GOLD stages, %			
1	33.3		
2	56.1		
3	10.5		
GOLD categories, %			
A	35.2		
B	44.4		
C	9.3		
D	11.1		
GINA steps, %			
1		3.8	
2		11.4	
3		32.9	
4		40.5	
5		11.4	
Median (IQR) FEV1, L	1.7 (1.3–2.2)	2.1 (1.8–3.1)	0.0001
Mean (SD) FEV1, %	71.7 (19.1)	88.6 (23.5)	< 0.0001
Median (IQR) FVC, L	3.3 (2.6–3.8)	3.3 (2.6–4.1)	0.82
Mean (SD) FVC, %	98.0 (17.2)	100.8 (20.7)	0.39
Mean (SD) FEV1/FVC	55.9 (10.1)	69.6 (10.4)	< 0.0001
Median (IQR) blood leucocytes, mmc	6.7 (5.9–8.1)	6.9 (5.7–8.0)	0.96
Mean (SD) blood neutrophils, %	57.5 (9.2)	53.6 (9.1)	0.01
Mean (SD) blood lymphocytes, %	28.5 (8.9)	32.5 (8.1)	0.006
Median (IQR) blood eosinophils, %	2.9 (1.8–3.9)	3.6 (2.0–6.0)	0.047
Median (IQR) blood eosinophils,	210.6 (117.8–307.8)	238.5 (135.7–427.0)	0.12
Median (IQR) PCR (*n* = 28 VS. 21)	0.3 (0.1–0.7)	0.3 (0.1–0.6)	0.93
Median (IQR) sputum cells, × 104/ml	204.5 (81.2–330.0)	125.5 (61–320)	0.23
Median (IQR) sputum macrophages, %	16.1 (9.7–27.0)	18.9 (8.8–31.1)	0.35
Median (IQR) sputum neutrophils, %	74.8 (60.4–82.2)	58.4 (25.1–74.4)	< 0.0001
Median (IQR) sputum eosinophils, %	1.7 (0.8–3.2)	4.3 (1.0–24.3)	0.002
Median (IQR) sputum lymphocytes %	0.8 (0.6–1.7)	1.4 (0.7–2.0)	0.12
Median (IQR) sputum epithelial cells %	3.3 (1.4–6.4)	4.0 (1.7–8.2)	0.34
Inhaled corticosteroids (ICS), %	10.7	85.9	< 0.0001
Exacerbations previous year, yes/no n (%)	23 (40.4)	34 (38.2)	0.88
Median (IQR) number of exacerbations during the previous year	0 (0–1)	0 (0–1)	0.86

*OSAS* Obstructive sleep apnoea syndrome, *IQR* interquartile range, *SD* standard deviation, *PCR* C reactive protein, *FEV*_*1*_ forced expiratory volume in 1 s, *FVC* forced vital capacity, *ICS* inhaled corticosteroids

**Table 2:** Comorbidities in COPD and asthmatic patients

Variables	COPD (*n* = 57)	Asthma (*n* = 89)	*P*-values
OSAS, n (%)	15 (26.3)	19 (21.3)	0.56
Obesity, n (%)	19 (33.3)	20 (22.5)	0.192
Hypertension, n (%)	30 (52.6)	29 (32.6)	0.03
Ischemic heart disease, n (%)	14 (24.6)	1 (1.1)	< 0.0001
Atrial fibrillation, n (%)	12 (21.1)	4 (4.5)	0.03
Diabetes mellitus, n (%)	5 (8.8)	8 (9.0)	1.0

*OSAS* obstructive sleep apnoea syndrome

No statistically significant differences were found between asthma and COPD in the frequency of: OSAS, obesity and diabetes. Diabetes, neoplasia, and bronchiectasis showed a low prevalence both in COPD and asthmatic patients and not considered for further evaluations.

### Correlation between blood and sputum eosinophils

**Fig. 1:**
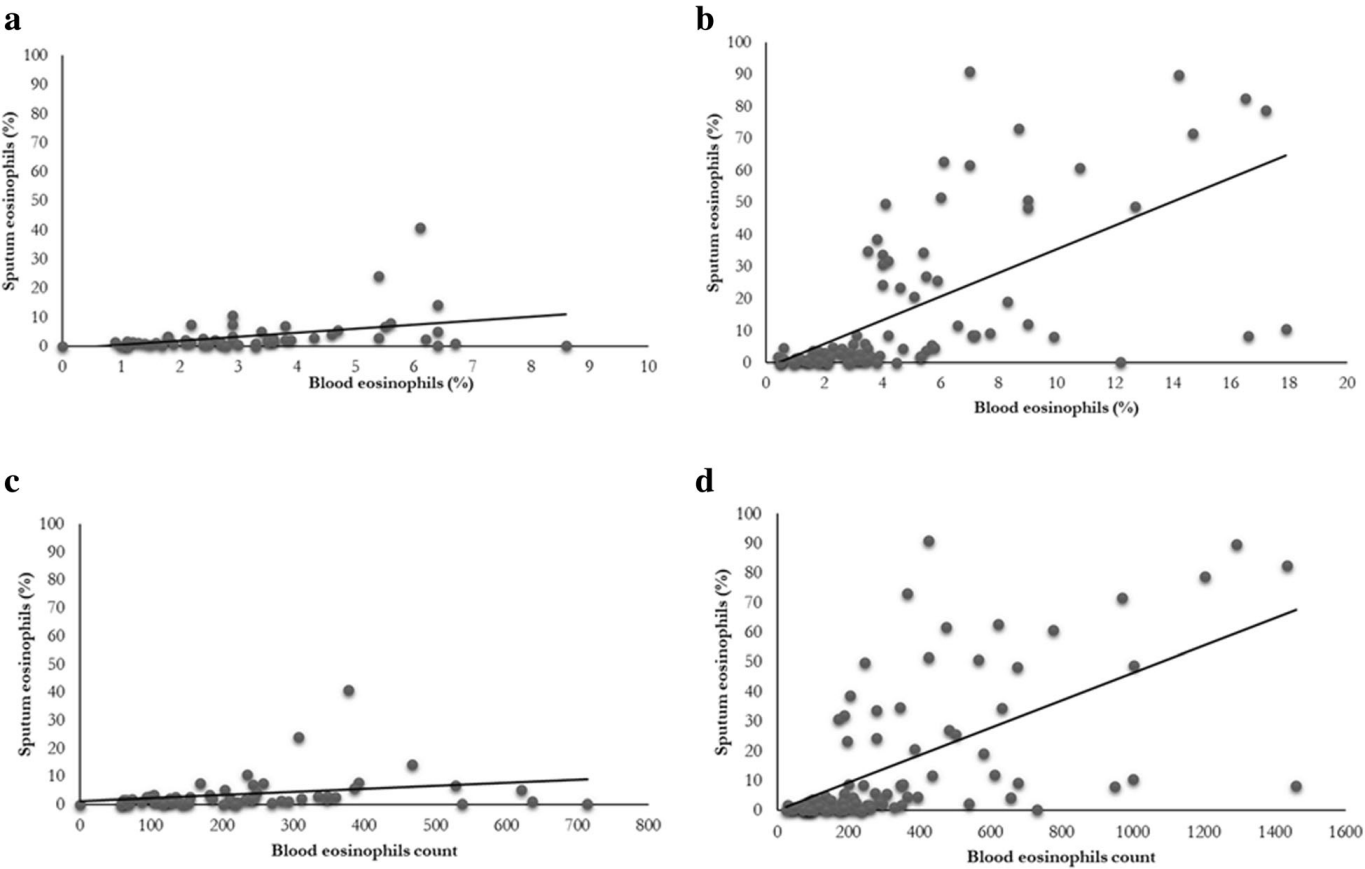
Correlation between sputum and blood eosinophils expressed as percentage (**a**-**b**) or absolute count (**c**-**d**) in COPD (**a**-**c**) and asthma (**b**-**d**)

Blood and sputum eosinophils expressed as percentages were correlated in COPD (rho = 0.40; *p* = 0.004), but less if compared with asthmatic subjects (rho = 0.71; P < 0.0001), Fig. 1.

When we expressed blood eosinophils as counts the correlation was slightly lower than that expressed as percentages in COPD (rho = 0.35; *p* = 0.01) and in asthmatic patients (rho = 0.68; *p* < 0.0001), Fig. 1.

We then divided COPD and asthmatic patients according to median values of blood eosinophils (both expressed as percentage and as count). In COPD patients, only when blood eosinophils were lower than the median value (210.6 eos/μl), blood and sputum eosinophils slightly correlated. In asthmatic patients, the correlation was consistently present and better when blood eosinophil count was higher than the median value, Table 3. Other cut-offs considered did not reveal stronger correlations in COPD patients (data not shown).

**Table 3:** Correlation between blood and sputum eosinophils in COPD and asthmatic subjects divided according to median blood eosinophil value (both percentage and count)

	rho (*p*-value)	rho (*p*-value)
COPD	Blood eosinophils < 2.9%	Blood eosinophils ≥2.9%
Blood eosinophils % and sputum eosinophils %	0.24 (0.22)	0.12 (0.53)
Blood eosinophils count and sputum eosinophils %	0.17 (0.40)	0.05 (0.78)
COPD	Blood eosinophils count < 210.6	Blood eosinophils count ≥210.6
Blood eosinophils % and sputum eosinophils %	0.45 (0.02)*	0.18 (0.35)
Blood eosinophils count and sputum eosinophils %	0.35 (0.07)	0.16 (0.42)
Asthma	Blood eosinophils < 3.6%	Blood eosinophils ≥3.6%
Blood eosinophils % and sputum eosinophils %	0.36 (0.02)*	0.37 (0.01)*
Blood eosinophils count and sputum eosinophils %	0.35 (0.02)*	0.37 (0.01)*
Asthma	Blood eosinophils count < 238.5	Blood eosinophils count ≥238.5
Blood eosinophils % and sputum eosinophils %	0.40 (0.007)*	0.48 (0.0007)*
Blood eosinophils count and sputum eosinophils %	0.28 (0.06)	0.48 (0.001)*

* *P* < 0.05

**Fig. 2:**
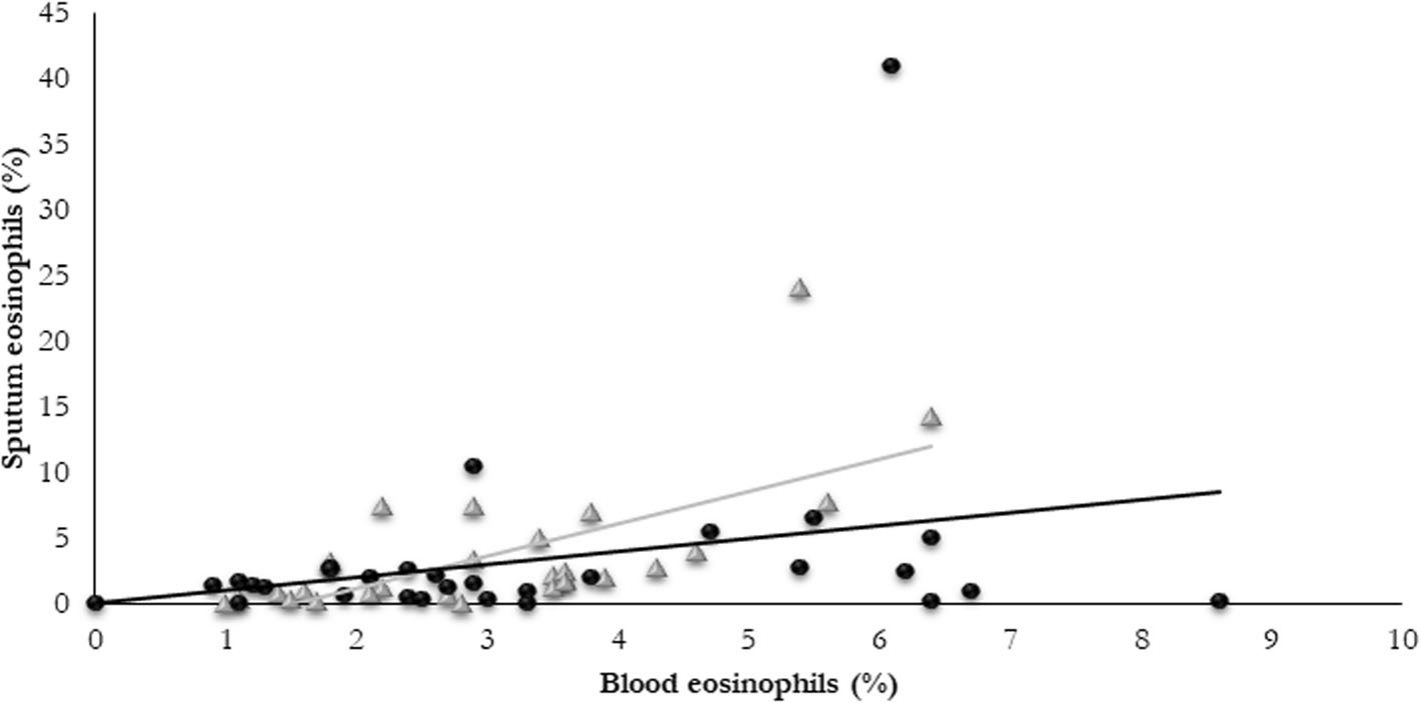
Correlation between sputum and blood eosinophils expressed as percentage in COPD patients with (circles) and without hypertension (triangles)

There was no correlation between blood and sputum eosinophils in COPD patients co-diagnosed with ischemic heart disease, atrial fibrillation or hypertension, Table 4. In Fig. 2 we reported the correlation between blood and sputum eosinophils in COPD subjects with or without hypertension. Furthermore, as most of the patients with ischemic heart disease and atrial fibrillation also had hypertension, we cannot conclude on the single effect of these cardiac comorbidities regarding the correlation between blood and sputum eosinophils.

**Table 4:** Correlation between blood and sputum eosinophils according to the presence of more frequent comorbidities

Comorbidity	No	Yes
COPD	rho (p-value)	rho (p-value)
OSAS (*n* = 42 VS. 15)	0.42 (0.006)	0.51 (0.05)
Obesity (*n* = 38 VS. 19)	0.44 (0.006)	0.46 (0.05)
Hypertension (*n* = 27 VS. 30)	0.72 (< 0.0001)	0.21 (0.28)
Ischemic heart disease (*n* = 43 VS. 14)	0.52 (0.0004)	0.22 (0.46)
Atrial fibrillation (*n* = 45 VS. 12)	0.53 (0.0002)	0.11 (0.74)
Asthma	No	Yes
OSAS (*n* = 70 VS. 19)	0.71 (< 0.0001)	0.73 (0.0004)
Obesity (*n* = 69 VS. 20)	0.71 (< 0.0001)	0.77 (0.0001)
Hypertension (*n* = 60 VS. 29)	0.77 (< 0.0001)	0.61 (0.0004)

*OSAS* obstructive sleep apnoea syndrome

Sex did not influence the correlation between blood and sputum eosinophils in any of the patients considered. No correlation between age and blood eosinophils was found in COPD and asthmatic patients, however, in COPD patients ≥73 years (median age) percentages of blood and sputum eosinophils were not correlated (rho = 0.22; *p* = 0.24).

No difference in the prevalence of ischemic heart disease and hypertension was found in COPD patients younger and older than 73 years (*p* = 0.54 and *p* = 0.48, respectively), while a tendency to a higher prevalence of atrial fibrillation was found in patients older than 73 years (*p* = 0.05).

In the asthmatic patients enrolled hypertension did not affect correlation between blood and sputum eosinophils, while ischemic heart disease and atrial fibrillation were too infrequent to evaluate their role.

In COPD patients with ischemic heart disease there was a tendency towards increased blood eosinophils compared to patients without this comorbidity (3.4%, 2.9–5.6 and 2.7%, 1.6–3.8) but the difference was not statistically significant (*p* = 0.11). In COPD patients, co-diagnosis with hypertension and/or atrial fibrillation was not associated with an increase of blood eosinophils.

No differences were found for sputum eosinophils in COPD patients with or without comorbidities.

When subjects were divided according to the reported exacerbations in the year before the evaluation, in COPD patients the correlation was quite similar (no exacerbations: rho = 0.42, *P* = 0.01 *n* = 34; exacerbations≥1: rho 0.42, *P* = 0.05, *n* = 23). In the asthmatic patients the correlation was higher in the group who reported ≥1 exacerbations (no exacerbations: rho = 0.61, *P* < 0.0001 *n* = 53; ≥1 exacerbations: rho 0.85, P < 0.0001, n = 34).

A few COPD patients were treated with ICS (*n* = 6, 2 associated with LABA and 4 with LABA and LAMA). ICS treatment did not affect correlation between blood and sputum eosinophils in asthmatic patients (*n* = 68/89, rho = 0.63; *P* < 0001).

## Discussion

The present study assessed the correlation between blood and sputum eosinophils in stable COPD patients in comparison with stable asthmatic patients. The percentage of blood eosinophils correlated with the percentage of sputum eosinophils to a lesser extent in COPD than in asthmatic patients. The percentage of blood eosinophils seems to reflected sputum eosinophilic inflammation slightly better than count. To the best of our knowledge, this is the first study in which the correlation between blood and sputum eosinophils was concurrently evaluated in COPD and asthmatic patients. We also found that in COPD patients there was no correlation between blood and sputum eosinophils when COPD subjects were older (≥73 years), had high blood eosinophils (≥ the median value) or in subjects co-diagnosed with ischemic heart disease, atrial fibrillation or hypertension. However, since ischemic heart disease and atrial fibrillation are often present with hypertension, we cannot conclude on the role of these comorbidities. Diabetes can be another comorbidity, which could affect the correlation between blood and sputum eosinophils, but its frequency was too low in the evaluated subjects. Most of the COPD patients enrolled in this study were not treated with ICS, whereas in the asthmatic group, the treatment with ICS did not affect the correlation between blood and sputum eosinophils.

Blood eosinophils expressed both as percentage and count have been recently used as surrogate marker of airway inflammation in asthma and in COPD. While in asthmatic patients this correlation, even if not strong, is convincing, in COPD patients more scepticism has been raised. We know that the strength of correlation between blood and sputum eosinophils varies in different studies both in asthma and in COPD, but to the best of our knowledge, none of them evaluated the correlation in the two groups of patients in the same study.

Blood eosinophils in COPD patients should be detected for therapeutic choices: to select patients to be treated with combination of ICS/bronchodilators is needed to reduce the risk of pneumonia in patients without an eosinophilic inflammation, and to evaluate possible therapies with monoclonal antibodies against IL-5 or its receptor, decreasing T2 inflammation. Increased blood eosinophils could represent a treatable trait in COPD patients, but it is not clear if this increase only reflects airway eosinophilia or more likely a mixed systemic and peripheral inflammation.

Concrete evidence that the correlation between blood and airway eosinophils in COPD was weak or even absent came from the study of Turato et al. where eosinophils in tissue biopsies did not reflect the amount of blood eosinophils [5]. Biopsies were obtained by central, peripheral airways and lung parenchyma. Only a correlation between eosinophils in lung parenchyma and in central airways was found.

Hartjes et al. in COPD patients not treated with ICS found a weak correlation between blood eosinophils and sputum, biopsies and BAL eosinophils [10]. However, the authors highlighted that due to the high variance, the prediction of airway eosinophilia through blood eosinophils is unreliable.

Schleich et al. found that in stable COPD patients with different treatments, part without ICS and part with ICS or oral corticosteroids, the best cut-off which reflects sputum eosinophils ≥3% was 215 eosinophils/μl (AUC 0.76, sensitivity 60% and specificity 93%) or 2.3% blood eosinophils (AUC of 0.7, sensitivity 62% and specificity 94%) [11]. In our study 22/57 subjects had blood eosinophils higher than 215 eosinophils/μl but only 10 of them had sputum eosinophils ≥3% (45%) and 29/57 had blood eosinophils higher than 2.3% but only 12 of them had sputum eosinophils ≥3% (41%).

Kolsum et al. [12] showed that COPD patients with high blood eosinophils have increased eosinophils in different lung sites, excluding subjects with intermediate blood eosinophils (between 150 and 250 eos/μl).

Eosinophils are produced by bone marrow and migrate to tissues attracted by different cytokines and chemokines. They can present different phenotypes sometimes protective and sometimes detrimental.

Changes in tissue metabolic state, stem cell activity, morphogenesis and regeneration are processes where eosinophils can be involved [13]. Eosinophils can reduce inflammation-related tissue damage [14], in line with reported data of decreased mortality in COPD patients with high blood eosinophils [15].

The presence of different subtypes of eosinophils with different roles and characterized by different membrane molecules demonstrated the heterogeneity of these cells, which were previously only described as steady state or activated cells [13]. Mesneil C at al., demonstrated at least in an animal model of asthma, that resident and inflammatory eosinophils were detected in blood, showing that their differentiation precedes their extravasation into tissues [16]. Therefore, blood eosinophils are a mix of heterogeneous cells with different roles and probably only a part of them are those recruited in the airways, particularly in the presence of complex systemic comorbidities.

COPD patients frequently have one or more comorbidities. COPD patients with hypertension are highly prevalent in our study and in these subjects, blood and sputum eosinophils are less or not correlated compared with those without this comorbidity.

Blood eosinophils, particularly in COPD patients, could increase because of systemic inflammation, cardiac heart failure or they could reflect an attempt to regulate perivascular adipose tissue and hypertension.

Recently it has been demonstrated that eosinophils play a pivotal role in metabolic homeostasis [16, 17] and in the maintenance of healthy perivascular adipose tissue functionality through the release of catecholamines, which mediate nitric oxide and adiponectin signalling [18]. Furthermore, eosinophil-deficient mice with hypertension recovered upon eosinophil reconstitution [18].

High serum triglyceride, low serum high-density lipoprotein cholesterol and chronic kidney disease were associated with high absolute eosinophil count [19, 20].

In asthmatic patients with hypertension, the correlation between blood and sputum eosinophils was not affected, probably because the trigger that attracts cells might overcome the systemic increase of eosinophils due to other causes.

Blood eosinophils exert not only a protective but also an inflammatory role. Sweetnam PM et al. found that increased blood eosinophil count is a risk factor for coronary heart disease [21] and Tanaka M et al. that eosinophil count is positively correlated with coronary artery calcification [22]. Recently it has been reported that increased absolute eosinophil count was independently associated with the presence of complex aortic arch plaques [23]. Eosinophil proteins activate platelets and promote thrombus formation [24]. Subjects with Churg Strauss syndrome, characterized by high blood eosinophils, present an increased incidence of atypical thrombotic events [25] and subjects with hypereosinophilic syndrome can present life-threatening disseminated thrombosis [26], probably due to eosinophil granule proteins, which favour hypercoagulability.

Recently a new unexpected role for eosinophils in haemostasis and thrombosis in response to vascular injury has been described [27]. It might not be surprising that in COPD patients, characterized by different comorbidities associated with eosinophil increase, blood eosinophils could be the result of cells coming from the bone marrow recruited for different mechanisms.

The effect of comorbidities on blood eosinophils and on the correlation between blood and airway eosinophils has scarcely been evaluated. DiSantostefano RL et al., found in a cohort of COPD patients that blood eosinophils increased in older male subjects with contemporarily severe asthma [28]. In our study, none of the COPD patients had a previous history of asthma, age was not correlated with blood eosinophils but in older COPD patients, age affected the correlation between blood and sputum eosinophils. They also found that among subjects with normal lung function, increased blood eosinophils were associated with congestive heart failure. We found that in COPD patients, ischemic heart disease, atrial fibrillation and hypertension can affect the correlation between blood and sputum eosinophils, suggesting that different triggers, other than airway inflammation, could increase blood eosinophils. More data are needed, particularly to evaluate the exclusive role of hypertension and cardiac comorbidities in a larger cohort of subjects.

In non-smoking COPD patients any percentage of blood eosinophils is predictive of a response to ICS in terms of reduction of exacerbation [29], while triple therapy significantly reduced the exacerbation rate compared with ICS/LABA in COPD patients with eosinophils of at least 2% [30]. Results obtained with the use of biologics in COPD patients are still controversial [31, 32].

## Conclusion

Blood and sputum eosinophils in COPD patients did not correlate as well as in asthmatic patients, probably due to blood eosinophils triggered in blood from bone marrow by different causes other than airway inflammation. An accurate clinical and biological characterization of COPD patients could help in understanding the role of blood eosinophils in COPD patients and using them as useful biomarker.

## Data Availability

Raw data are available upon request to the corresponding author.

## Abbreviations

AUC: Area under the curve
BAL: Bronchoalveolar Lavage
BMI: Body Mass Index
COPD: Chronic Obstructive Pulmonary Disease
FEV_1_: Forced expiratory volume in the 1st second
FVC: Forced vital capacity
GINA: Global Initiative for asthma
GOLD: Global Initiative for Chronic Obstructive Lung Disease
ICS: Inhaled Corticosteroids
IQR: Interquartile range
LABA: Long-acting β2 agonists
LAMA: Long-acting muscarinic antagonists
OSAS: Obstructive Sleep Apnoea Syndrome
PCR: C-reactive protein
SD: Standard Deviation
T2 inflammation: T lymphocyte type 2 inflammation
